# *In Silico* Discovery of Potential Uridine-Cytidine Kinase 2 Inhibitors from the Rhizome of *Alpinia mutica*

**DOI:** 10.3390/molecules21040417

**Published:** 2016-04-08

**Authors:** Ibrahim Malami, Ahmad Bustamam Abdul, Rasedee Abdullah, Nur Kartinee Bt Kassim, Peter Waziri, Imaobong Christopher Etti

**Affiliations:** 1 MAKNA-Cancer Research Laboratory, Institute of Bioscience, Universiti Putra Malaysia, 43400 Serdang, Selangor, Malaysia; petermwaziri@gmail.com; 2 Department of Veterinary Pathology and Microbiology, Faculty of Veterinary, Universiti Putra Malaysia, 43400 Serdang, Selangor, Malaysia; rasedee@gmail.com; 3 Department of Chemistry, Faculty of Science, Universiti Putra Malaysia, 43400 Serdang, Selangor, Malaysia; kartinee@upm.edu.my; 4 Department of Pharmacology and Toxicology, Universiti Putra Malaysia, 43400 Serdang, Selangor, Malaysia; ettimaobong@gmail.com

**Keywords:** UCK2, *in silico*, flavokawain B, alpinetin, *Alpinia mutica*, nucleoside analogues, amino acid active site residues

## Abstract

Uridine-cytidine kinase 2 is implicated in uncontrolled proliferation of abnormal cells and it is a hallmark of cancer, therefore, there is need for effective inhibitors of this key enzyme. In this study, we employed the used of *in silico* studies to find effective UCK2 inhibitors of natural origin using bioinformatics tools. *A*n *in vitro* kinase assay was established by measuring the amount of ADP production in the presence of ATP and 5-fluorouridine as a substrate. Molecular docking studies revealed an interesting ligand interaction with the UCK2 protein for both flavokawain B and alpinetin. Both compounds were found to reduce ADP production, possibly by inhibiting UCK2 activity *in vitro*. In conclusion, we have identified flavokawain B and alpinetin as potential natural UCK2 inhibitors as determined by their interactions with UCK2 protein using *in silico* molecular docking studies. This can provide information to identify lead candidates for further drug design and development.

## 1. Introduction

Nucleotides are the foundation of all physiological functions required for cell growth and replication and any genetic changes will therefore lead to disturbances in nucleotide pools [1,2]. In cancer cells, these genetic changes can show characteristic DNA/RNA modifications and activities of modifying enzymes, that results in fluctuations in nucleotide levels [3]. In mammalian cell, RNA is in constant turned over in cells, both during the production of mature RNAs from longer precursors and to regulate the amounts of mRNA expressed in cells. During the metabolic breakdown of polymeric RNA and DNA, the resulting NMPs are released which can then be recycled by the action of the uridine-cytidine kinase 2 (UCK2) via alternative salvage pathway [4]. Drugs such 5-fluorouracil and hydroxyurea inhibit *de novo* nucleotide biosynthesis. However, inhibition of *de novo* pathway alone is insufficient to produce effective treatment, as such, unutilized nucleotides are salvaged via pyrimidine synthesis.

UCK2 is an enzyme that catalyzes the phosphorylation of uridine and cytidine nucleotides to their corresponding uridine monophosphate (UMP) and cytidine monophosphate (CMP) [5]. Phosphorylation of UMP and CMP are an essential requirement to form 5′-triphosphate nucleotides required for gene synthesis. UCK2 have been reported to be expressed only in the placenta and its over expression have been implicated in several rapidly proliferating cancer cells [6,7]. Therefore, the selective expression of UCK2 in cancer cells makes it a potential target for cancer chemotherapy.

Nucleoside analogues are being used to treat different cancer cells via their phosphorylation catalyzed UCK2. Such nucleoside analogues under investigation in clinical trials either as a single drug entity or in combination with other cytotoxic agents includes 1-(3-*C*-ethynyl-beta-d-ribo-pentofuranosyl)cytosine (ECyd, TAS 106) [8,9] and fluorocyclopentenylcytosine (RX-3117) [10]. These nucleoside analogues depend on UCK2 for phosphorylation to their triphosphate form to exert their pharmacological activity and once phosphorylated, the nucleoside analogues interfere with the synthesis of RNA or DNA which are vital cellular processes required for growth and development [6]. Hence, TAS 106 use is accompanied by several serious toxicity issues such as neurotoxicity, neutropenia, febrile neutropenia, pneumonia, leukopenia, thrombocytopenia, tremor, pain, hyperesthesia, asthenia, anorexia, nausea, vomiting, myelosuppression, as well as dermatological effects [8,9,11,12,13].

Low molecular weight natural products are capable of inhibiting enzyme catalytic activity due to the remarkable complexity of their chemical structures. Some natural anticancer agents used nowadays exert their pharmacological action by inhibiting a human enzyme, particularly ones involved in metabolic pathways [14]. Scientists have for long focused only on the design and synthesis of drugs that are UCK2 dependent, hence, forgetting that bioactive natural products which are capable of reducing or completely inhibiting enzyme catalytic activity can be used as a chemotherapeutic targeting UCK2 activity. Due to the serious side effects evidenced by these ribonucleoside analogues, effective drugs of natural origin with less potential side effect to patients would be highly desirable. To date, there has not been any research on any single natural bioactive compound targeting the UCK2 enzyme and no inhibitors of this enzyme have been so far reported. In our search for natural bioactive compounds targeting UCK2, we report here for the first time using *in silico* visual screening that flavokawain B and alpinetin inhibit UCK2.

## 2. Results and Discussion

### 2.1. Cell Viability Studies

**Figure 1:**
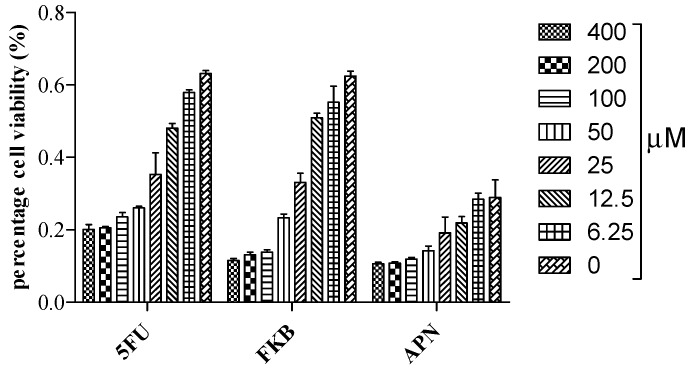
Percentage cell viability of HT 29 cells treated with 5FU, FKB, and APN. MTT assay was used to determine the IC_50_ of the drugs at different concentrations in µM for 72 h.

To determine the percentage cell viability of HT 29 cells, the MTT colorimetric assay was used in this investigation to measure the amount of viable cells after 72 h of incubation. Flavokawain B (FKB) and alpinetin (APN) inhibit 50% cell proliferation at an IC_50_ of 29.84 µM (8.47 µg/mL) and 48.58 µM (13.12 µg/mL), respectively (S1 files) (Figure 1). 5-Fluorouracil (5FU) was used as positive control in this study. The inhibition of proliferating cells by FKB at a very low concentration has previously been reported in colon cancer [15,16].

### 2.2. Molecular Docking Studies

#### 2.2.1. Redocking Analysis

Autodock is an effective tool used to obtain unbiased docking of flexible inhibitors in enzyme active sites [17]. Autodock 4 uses a semiempirical free energy force field to predict the binding free energies of small molecules to macromolecular targets [18]. In order to validate our data sets using Autodock 4, a control docking was performed on UCK2 protein in complex with the inhibitor CTP using a root-mean-square deviation (RMSD) tolerance of 2.0 Å. The results of the redocking study are shown in Table 1. Autodock 4 successfully redocks the complex with the lowest energy conformation showing a RMSD of 0.829 Å from the reference structure. The lowest energy docked conformation has an estimated free binding energy of −14.27 kcal/mol, intermolecular energy of −16.66 kcal/mol, and inhibition constant (Ki) of 34.63 pM. The obtained RMSD of less than 2.0 Å confirmed the validity of our docking data sets (S2 files).

#### 2.2.2. Docking Analysis of FKB and APN on UCK2 Protein

FKB was estimated to have a lowest binding energy of −8.47 and intermolecular energy of −10.26 kcal/mol, and inhibition constant (Ki) of 618.12 nM (Table 1). On the other hand, alpinetin had −8.86 binding and −9.75 kcal/mol, intermolecular energy of, and a Ki of 321.38 nM. The intermolecular hydrogen interactions between the amino acid residues in the catalytic active site of UCK2 and its inhibitors are tabulated in Table 2.

**Figure 2:**
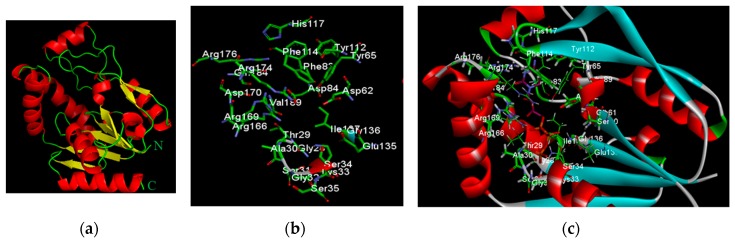
*In silico* redocking of UCK2 protein in complex with inhibitor CTP. (**a**) Complete x-ray structure of UCK2 receptor protein shown as a cartoon; (**b**) Amino acid residues in the active site of UCK2; (**c**) Interactions of CTP with UCK2 as identified by *in silico* docking analysis. CTP shown in ball-and-stick model with purple indicating carbon atoms, white for hydrogen, blue for nitrogen, red for oxygen, and phosphorus in orange.

**Figure 3:**
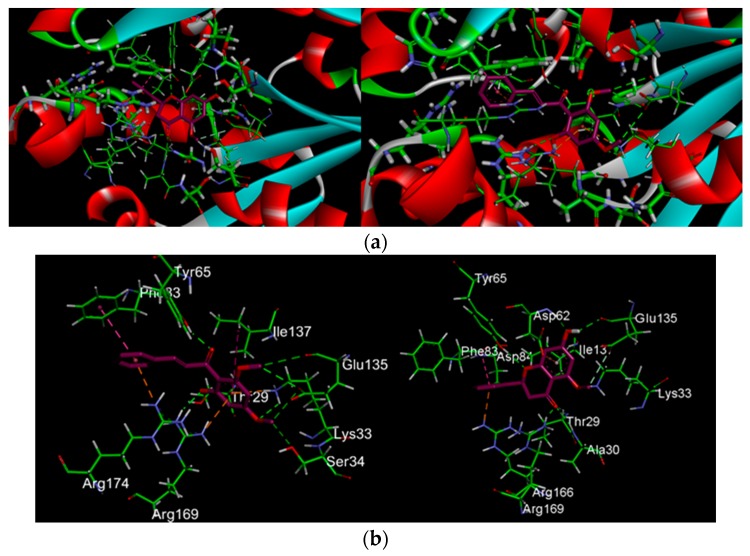

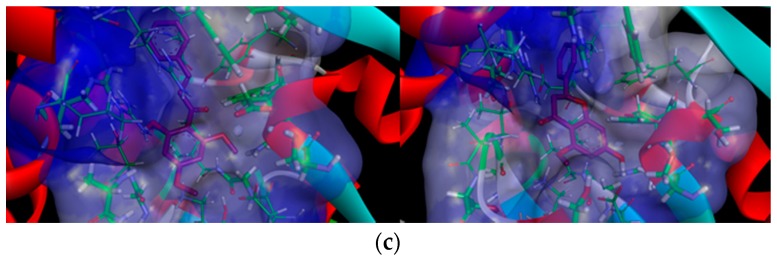
(**a**) Interactions of ligands with UCK2 protein as identified by *in silico* docking analysis; (**b**) Ligands interacting with the amino acids residues in the active sites of UCK2 protein; (**c**) Surface representation of the hydrophobic contacts of bound ligand and the ligand binding pocket of UCK2 protein shown as translucent blue surface. Ligands shown as a ball-and-stick model with purple indicating carbon atoms, white for hydrogen, and red for oxygen Hydrogen bonds shown in green dotted lines, electrostatic in yellow, and hydrophobic in purple.

Figure 2 and Figure 3 show a total of eight hydrogen bond interactions formed between the catalytic active site of UCK2 protein and flavokawain B as well as alpinetin, respectively. The hydrogen and oxygen of the 4-methoxy group of flavokawain B form hydrogen bonds with the Oγ atom of Ser-34 and Hζ2 atom of Lys-33, while the 2-hydroxyl group forms a hydrogen bond with the Hη22 atom of Arg-169. These amino acid residues form the binding site for the γ- and β-phosphate moieties of ATP. ATP has been shown to adopt a pentacoordinate transition state and produce an anionic charge upon nucleophilic attack by the 5′-oxygen atom at the γ-phosphate moiety of ATP and the side chains of Lys-33, Arg-169 and Arg-174 are the potential candidates to stabilize this anionic charge [19]. Apart from the γ- and β-phosphate moieties of ATP, flavokawain B also forms three important hydrogen bonds with Glu-135 involved in coordination with a magnesium ion (Mg^2+^). A Mg^2+^ is found to play a crucial role in the stabilization of the transition state by its coordination with the γ- and β-phosphate oxygens of ATP [7,19]. Other hydrogen bonds formed between flavokawain B and the amino acid side chains of UCK2 protein involve the 2-hydroxyl group and 9′ ketone group of flavokawain B with Oγ1 and the Hη atom of Thr-29 and Tyr-65, respectively.

**Figure 4:**
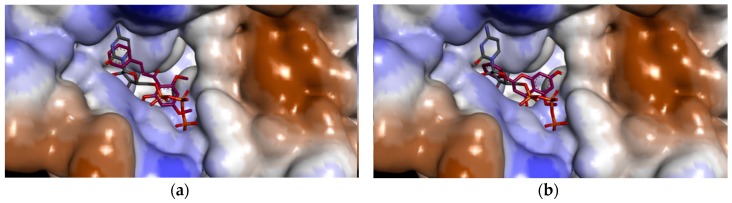
Comparison of the binding mode of UCK2 inhibitors in the ligand binding pocket of UCK2 surface. (**a**) FKB shown in purple color and CTP in maroon color; (**b**) APN shown in purple color and CTP in maroon color.

On the other hand, like flavokawain B, alpinetin also binds to the γ- and β-phosphate moieties of ATP (S2 files). Three hydrogen bonds are formed with the amino acid side chains of HN, Hζ1 and Hη12 atom of Ala-30, Lys-33 and Arg-169, respectively. The Oε1 atom and carboxyl group of Glu-135 also form two hydrogen bonds with the 5-hydroxyl group and methoxy group of alpinetin. In addition, three hydrogen bonds are also formed with the side chain of Thr-29, Tyr-65 and Asp-62. Asp-62 has been known to activate the 5′-hydroxyl group of ribonucleosides because it’s the only amino acid residue that can function as a catalytic base around the 5′-hydroxyl group [7,19]. Therefore, the hydrogen bond between the hydroxyl group of alpinetin and HN atom of Asp-62 may strongly inhibit the 5′-hydroxyl group interaction of ribonucleosides, even if they were phosphorylated. In comparison to the control feedback inhibitor CTP, the binding mode of UCK2 inhibitors positioned in such a way that the inhibitors buried themselves deep in the ATP binding pocket surrounded by amino acid side chains of the ATP binding site (Figure 4). 

It has been shown that ligand binding at both the phosphate donor and acceptor binding sites competitively inhibit UCK [19]. Therefore, the inhibitors are bound to the site where γ- and β-phosphate moieties of ATP bind, thus, the inhibition may be competitive. Flavokawain B and alpinetin are therefore estimated to inhibit UCK2 protein by binding to the catalytic active site of ATP, thus inhibiting ATP from binding to its active site in the UCK2 protein.

### 2.3. In Vitro Kinase Activity

**Figure 5:**
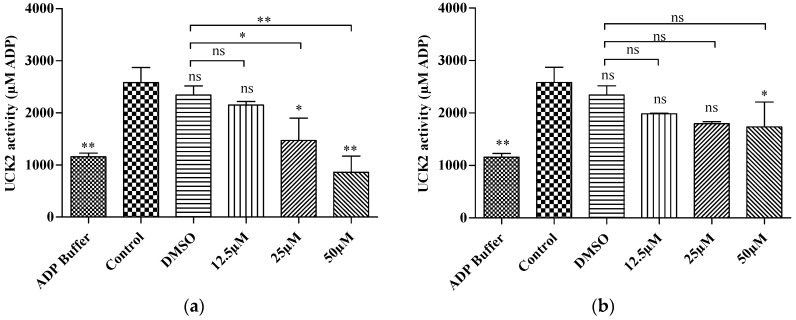
UCK2 enzyme activity was measured based on the amount of ADP produced from the enzyme reaction (**a**) Cell lysate incubated in the presence of FKB (**b**) Cell lysate incubated in the presence of APN. Florescence intensity (λ_ex_ = 450 nm/λ_em_ = 590) was monitored using a fluorescence plate reader. Results were expressed as mean ± SD of three independent experiments, * *p* < 0.05, ** *p* < 0.01, ns: non-significant when compared to the control.

The fluorimetric kinase assay is a non-radioactive determination of kinase activity based on the amount of ADP produced by an enzyme reaction in the presence of ATP, where the amount of ADP formed is directly proportional to the enzyme activity. To validate our molecular docking result, we carried out a preliminary kinase activity by measuring the amount of ADP produced by utilizing 5-fluorouridine as a substrate in the presence of either FKB or APN. Interestingly, both compounds reduced enzyme activity in a dose-dependent manner (S3 files, Figure 5). A significant reduction in enzyme activity at a concentration of 25 µM (7.1 µg/mL) and 50 µM (14.2 µg/mL) was observed in a dose-dependent manner when incubated with FKB. A significant difference was also observed in a dose-dependent manner compared to DMSO treated control. This shows that the solvent (DMSO) used as vehicle does not contribute significantly to the enzyme activity since enzyme assays can tolerate high DMSO concentrations up to 5%–10% [20].

On the other hand, a significant reduction in enzyme activities was only observed when incubated with 50 µM (13.5 µg/mL) of APN, but this shows no significant difference compared to DMSO treated control. Thus, the potent enzyme inhibition of APN is about 2-fold lower compared to that of FKB at 25 µM. Research has shown that UCK2 also phosphorylates uridine nucleoside analogs such as 5-fluorouridine. The percentage efficiency of phosphorylation of 5-fluorouridine have been shown to be more than 100% efficiency when correlated to the efficiency of uridine phosphorylation catalyzed by UCK2 [6]. The results of the current investigation shown that 5-fluoroudine may have been phosphorylated by the presence of UCK2 in the untreated cell lysate and the enzyme may have been inhibited in HT 29 cells treated with either FKB or APN.

## 3. Materials and Methods

### 3.1. General Information

The following instruments were issued: Electrothermal IA 9100 series melting point apparatus (Staffordshire, UK), glass vacuum column, aluminium TLC plates (silica gel 60 F_254_, Merck Millipore, Darmstadt, Germany), Bruker Avance 500 MHz Nuclear Magnetic Resonance (NMR) spectrometer (Billerica, MA, USA). NMR spectra were recorded in CDCl_3_ (δ 7.26 as internal reference) and reported in ppm (δ), and coupling constants in Hertz, NMR peak patterns are described as broad (br), singlets (s), doublets (d), double-doublets (dd) triplets (t) and multiplets (m). GC-MS (Agilent Technologies, Santa Clara, CA, USA), fluorescence mucriplate reader (BioTek, Winnoski, VT, USA), ELISA microplate reader (Beckman, Brea, CA, USA). All other chemicals and reagents were of analytical grade and obtained from Sigma-Aldrich (St. Louis, MO, USA).

### 3.2. Plant Material

About 6 kg of the rhizomes of *Alpinia mutica* was collected from the Biodiversity Unit of the Institute of Bioscience, Universiti Putra Malaysia Serdang. The rhizomes were washed to remove extraneous matter, sliced into small pieces, and air-dried at room temperature. The dried rhizome was then ground into a fine powder using a grinding machine.

### 3.3. Extraction and Isolation of FKB and APN

**Figure 6:**
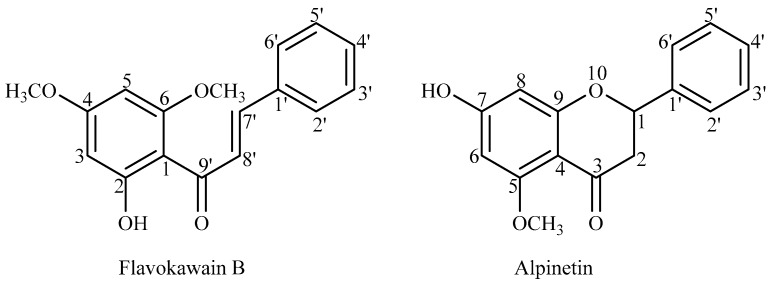
Skeletal structures of flavokawain B and alpinetin.

Extraction and isolation of FKB and ALP were performed based on the reported procedure [21] with some modifications. Ground plant material weighing 628.35 g was soaked and allowed to macerate at room temperature for 72 h sequentially with solvents of increasing polarity, first with hexane (3.2 L) and then chloroform (3.2 L) for three consecutive times. Each of the solutions obtained was filtered and concentrated in a rotatory evaporator at a reduced pressure to obtain a crude extract. The final concentrate was further allowed to dry at room temperature to complete dryness. Each of the crude fractions, *i.e.*, hexane (18.22 g, 2.90%) and chloroform (24.41 g, 3.88%) was was subjected to column chromatography for isolation and purification to obtain pure compounds using silica gel as stationary phase and mixtures of petroleum ether, ethyl acetate and methanol in various proportions as mobile phases. All the fractions were monitored by TLC, spotted under UV and the major spot identified by spraying with 10% H_2_SO_4_ and heated at 100 °C. The purified natural products were then analyzed by spectroscopic analysis, in particular, ^1^H- and ^13^C-NMR spectroscopy as well as direct infusion mass spectrometry (DIMS). Further washing of the hexane and chloroform fractions with petroleum ether afforded FKB (melting point 82–84 °C; 200 and 226.4 mg, 1.1% and 0.93%, respectively) while APN was obtained from the chloroform fraction after washing with petroleum ether (melting point 212–214 °C; 32.4 mg, 0.13%). Both FKB and APN (Figure 6) were identified and matched previously established NMR and mass spectral data (S4 files and S5 files) [22,23,24,25,26].

### 3.4. Cell Culture

The HT-29 cell line (ATCC) used in this study was obtained from the MAKNA Cancer Research Laboratory of the Institute of Bioscience. The cell line was sub-cultured in DMEM media (Sigma-Aldrich) supplemented with 10% fetal bovine serum (FBS) (PAA, Freibug, Germany) and 1% 100 IU penicillin and 100 µg/mL streptomycin (Sigma). The starting culture was at 1 × 10^4^ cells/mL and maintained at a temperature of 37 °C in a humidified incubator containing 5% CO_2_. Cultures were continuously maintained by routine trypsinization (0.05% Trypsin-EDTA) of cells at 70%–80% confluence.

### 3.5. Drugs

5-Fluorouracil and bioactive compounds isolated from rhizome of *Alpinia mutica* was used in this investigation. A stock solution of 5-fluorouracil/bioactive compound was prepared in a concentration of 400 µM in 50 µL dimethyl sulfoxide (DMSO) and the final concentration of DMSO will be 0.1% (*v*/*v*).

### 3.6. Cell Viability Assay

The effect of bioactive compounds on colorectal cancer was determined by a MTT assay. Briefly, HT 29 cells were seeded in 96-well microplates at a density of 0.5 × 10^4^ cells/mL. Cells were treated after 24 h incubation at different concentrations with FKB, APN or 5FU (400, 200, 100, 50, 25, 12.5, and 6.25 µM) in serial dilution for 72 h. After 72 h incubation, 20 µL of MTT stock solution (5 mg/mL) was added to each well and 100 µL of DMSO was added to each well after 4 h incubation in the dark. The amount of purple formazan formed was measured colorimetrically at 570 nm. Cell viability was expressed as the percentage of amount of viable cells to that of the amount of the total cell population and the potency of testing drugs to inhibit cell growth by 50% was expressed as IC_50_. The cell viability assay was carried out in three independent experiments.

### 3.7. Molecular Docking Simulation Studies

Molecular docking studies were performed using Autodock version 4.2 [18], analyzed and visualized using Discovery Studio visualizer version 4.5. The X-ray protein crystal structure (2.6 Å resolution) of human uridine-cytidine kinase 2 in complexed with a feedback inhibitor, CTP (1UDW.pdb ID) [19] was obtained from the Protein Data Bank (www.pdb.org). The three dimensional structures of FKB and APN was obtained from National Centre for Biotechnology information PubChem database [27] in an SDF file format and converted into the PDB file format using DS visualizer 4.5. Gasteiger charges were added to the ligand and all non-polar hydrogen atoms were deleted and their charges merged with the carbon atoms. The root of the molecule was detected, rotatable bonds were defined, and the number of torsions was set to 6. Prior to molecular docking, all solvent molecules, heteroatoms and the co-crystallized ligand (CTP) were removed from the structure [28,29] and all missing hydrogen atoms in the protein were added. The grid parameter file was prepared by setting the grid maps of 40 × 40 × 40 Å grid points in xyz, 0.375 Å spacing, and the grid box was positioned directly at the center on CTP-binding site of the crystal structure 1UDW (grid center 11.359 Å, 38.57 Å, and 33.111 Å in xyz-coordinates). Lamarckian genetic algorithm was used to carry out conformational searching in molecular docking simulation studies [18]. A molecular docking experiment was performed using 2,500,000 energy evaluations for 100 numbers of GA Runs per ligand with a population size set at 150.

### 3.8. Preparation of Cell Lysate

HT 29 cells were collected by trypsinization at 70%–80% confluence and centrifuged at 1000 rpm for 5 min at 4 °C. Supernatant was discarded, re-suspended in ice cold PBS and centrifuge at 1000 rpm for 5 min. Supernatant was discarded and the cell pellet was re-suspended in an appropriate volume of ProteoJET mammalian cell lysis reagent (Fermentas, Burlington, ON, Canada) and vortex for 5–10 s. The cells were allowed to incubate at room temperature on a shaker at approximately 900 rpm for 10 min and centrifuge at 18,000× *g* for 15 min. The supernatant was carefully collected, aliquots in PCR tubes and stored at −80 °C until use. Total protein concentration in the cell lysate using Bradford reagent (Bio-Rad Laboratories, Hercules, CA, USA).

### 3.9. In Vitro Kinase Activity Assay

The inhibitory activity of bioactive compounds on UCK2 were assayed using a Universal fluorimetric kinase assay kit (Sigma). A total 80 µL volume of the kinase reaction mixture was set up containing 20 µL of ADP Buffer, 25 µL of cell lysate/H_2_O, 20 µL of 12.5, 25, and 50 µM test drug/DMSO/H_2_O, 10 µL of 0.5 mM 5-fluorouridine/H_2_O, and 5 µL of 1 mM ATP/H_2_O. The reaction mixture was incubated in a water bath at 37 °C for 30 min. 20 µL of the kinase reaction was added into 96 black well microplate. For each well containing 20 µL kinase reaction, 20 µL of ADP sensor buffer was added, followed by 10 µL of the ADP sensor solution and the assay mixture was allowed to incubate for 15 min in the dark at room temperature. Florescent intensity (λ_ex_ = 450 nm/λ_em_ = 590) was monitored using a fluorescence plate reader. This assay was carried out in three independent experiments.

### 3.10. Statistical Analysis

The results of the experiments are reported as mean ± SD for at least three replicate analyses for each sample. Statistical analysis was performed using GraphPad Prism 5.0. Analyses of variance are performed using the ANOVA procedure followed by Dunnett’s and Bonferroni’s test for multiple comparison.

## 4. Conclusions

In conclusion, we have shown for the first time that flavokawain B and alpinetin act as potential UCK2 inhibitors *in silico*. Flavokawain B and alpinetin have shown interesting inhibition of this key enzyme involved in gene synthesis. This inhibition may provide information supporting their use as lead candidates for further drug development. Moreover, further studies are currently under way to determine the molecular mechanism of action by which FKB and ALP inhibit UCK2 from further phosphorylation of nucleosides during gene replication.

## Figures and Tables

**Table 1:** The lowest energy docked conformation from each ligand.

Ligand	Free Binding Energy *	Inhibition Constant, Ki	Intermolecular Energy *	vdW + Hbond + Desolv Energy *	Electrostatic Energy *
CTP	−14.27	34.63 pM	−16.66	−12.59	−4.07
FKB	−8.47	618.12 nM	−10.26	−9.96	−0.30
APN	−8.86	321.38 nM	−9.75	−9.04	−0.71

* kcal/mol; CTP: cytosine-5′-triphosphate, FKB: flavokawain B, APN: alpinetin.

**Table 2:** Intermolecular hydrogen interaction between UCK2 protein and its inhibitors.

UCK2	HB with CTP	HB with FKB	HB with APN
**Active Residues**	**(BD ≤ 3.5 Å, DHA ≥ 90°)**	**(BD ≤ 3.5 Å, DHA ≥ 90°)**	**(BD ≤ 3.5 Å, DHA ≥ 90°)**
Thr29	Hβ with 3′O	Oγ1 with 2 H	Hα with 3 O
Ala30	Hα with Oγ3, HN with Oα2		HN with 3 O
Gly32	HN with Oγ2		
Lys33	Hζ2 with Oγ2, HN with Oγ2, Hζ1 with Oα1, Hζ1 with Oβ3	Hζ2 with 4 O	Hζ1 with 5 O
Ser31	HN with Oγ2		
Ser34	HN with Oγ1, Hα with Oβ1, Hβ2 with Oγ1	Oγ with 4′ H	
Asp62			HN with 7 O
Tyr65		Hη with 9′ O	Hη with 10 O
Phe83			
Asp84	Oγ1 with 2′O, Oγ2 with 3′O		
Tyr112	Oη with 4N		
His117	Nδ1 with 4N		
Glu135		Oε1 with 10′ H, 4′ H	Oε1 with 5 H
		O with 10′ H	O with 7 H
Arg166	Oη12 with 3′O, Oη22 with 2′O, Oη22 with 3′O		
Arg169	Hη22 with Oγ2	Hη22 with 2 O	Hη12 with 3O
Arg174	Oη11 with 4′O		
Arg179			

HB: hydrogen bond, BD: bond distance, DHA: D, hydrogen donor; H, hydrogen; A, hydrogen acceptor.

## Supplementary Materials

Supplementary materials can be accessed at: http://www.mdpi.com/1420-3049/21/4/417/s1.
