# Supplementary material for: In Silico Discovery of Potential Uridine-Cytidine Kinase 2 Inhibitors from the Rhizome of Alpinia mutica
Source: Molecules. 2016 Apr 8;21(4):417. doi: 10.3390/molecules21040417 (PMC6274218; doi:10.3390/molecules21040417)

This report was created by ACD/NMR Processor Academic Edition. For more information go to www.acdlabs.com/nmrproc/

08/02/2016 14:49:32

Acquisition Time (sec)	1.0224	***Comment***	IB17 Ibrahim / Dr ahmad Bustamam in CDCl3		
Date	Jan 29 2016	***Date Stamp***	Jan 29 2016		
File Name	C:\Users\dfgdf\Desktop\NMR spec\ibrahim-13c\ibrahim_ib17_13c.fid\fid				
Frequency (MHz)	125.71	***Nucleus***	13C	***Number of Transients***	1216
Original Points Count	32768	***Points Count***	65536	***Pulse Sequence***	s2pul
Receiver Gain	54.00	***Solvent***	CHLOROFORM-d		
Spectrum Offset (Hz)	13826.9531	***Spectrum Type***	STANDARD	***Sweep Width (Hz)***	32051.28
Temperature (degree C)	25.000				

ibrahim_ib17_13c

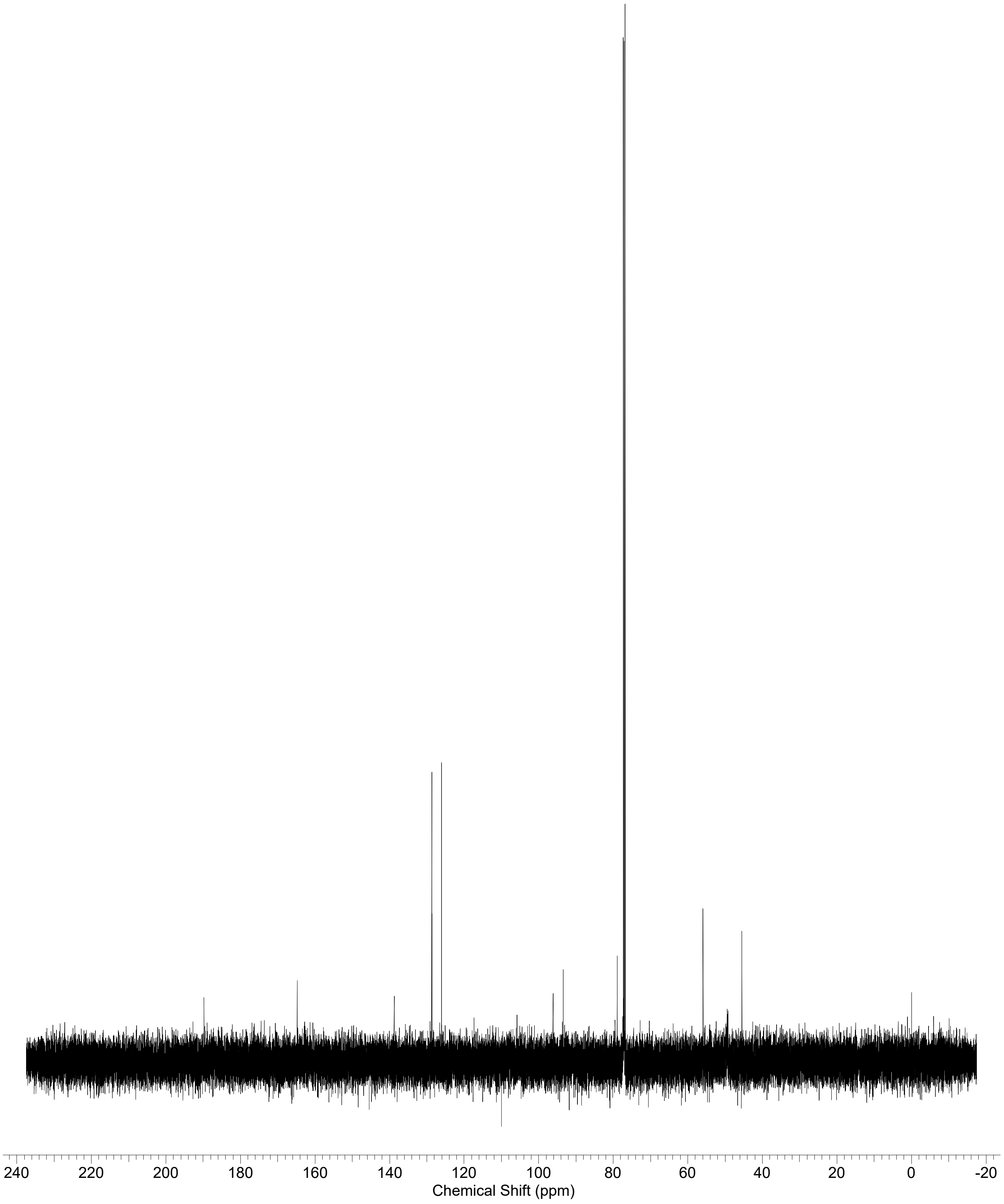

Supplement: Supplementary file 1 [file molecules-21-00417-s001.zip › Supplementary/S4 files/CNMR/APN.pdf]
