# Supplementary material for: In Silico Discovery of Potential Uridine-Cytidine Kinase 2 Inhibitors from the Rhizome of Alpinia mutica
Source: Molecules. 2016 Apr 8;21(4):417. doi: 10.3390/molecules21040417 (PMC6274218; doi:10.3390/molecules21040417)

This report was created by ACD/NMR Processor Academic Edition. For more information go to www.acdlabs.com/nmrproc/

10/02/2016 11:33:51

Acquisition Time (sec)	2.0447	*Comment*	IB17 Ibrahim / Dr ahmad Bustamam in CDCl3		
Date	Jan 15 2016	*Date Stamp*	Jan 15 2016		
File Name	C:\Users\dfgdf\Desktop\NMR spec\ibrahim_ib17_1h.fid\fid			*Frequency (MHz)*	499.90
Nucleus	1H	*Number of Transients*	16	*Original Points Count*	16384
Points Count	32768	*Pulse Sequence*	s2pul	*Receiver Gain*	22.00
Solvent	CHLOROFORM-d			*Spectrum Offset (Hz)*	3005.2056
Spectrum Type	STANDARD	*Sweep Width (Hz)*	8012.82	*Temperature (degree C)*	25.000

IB17.esp

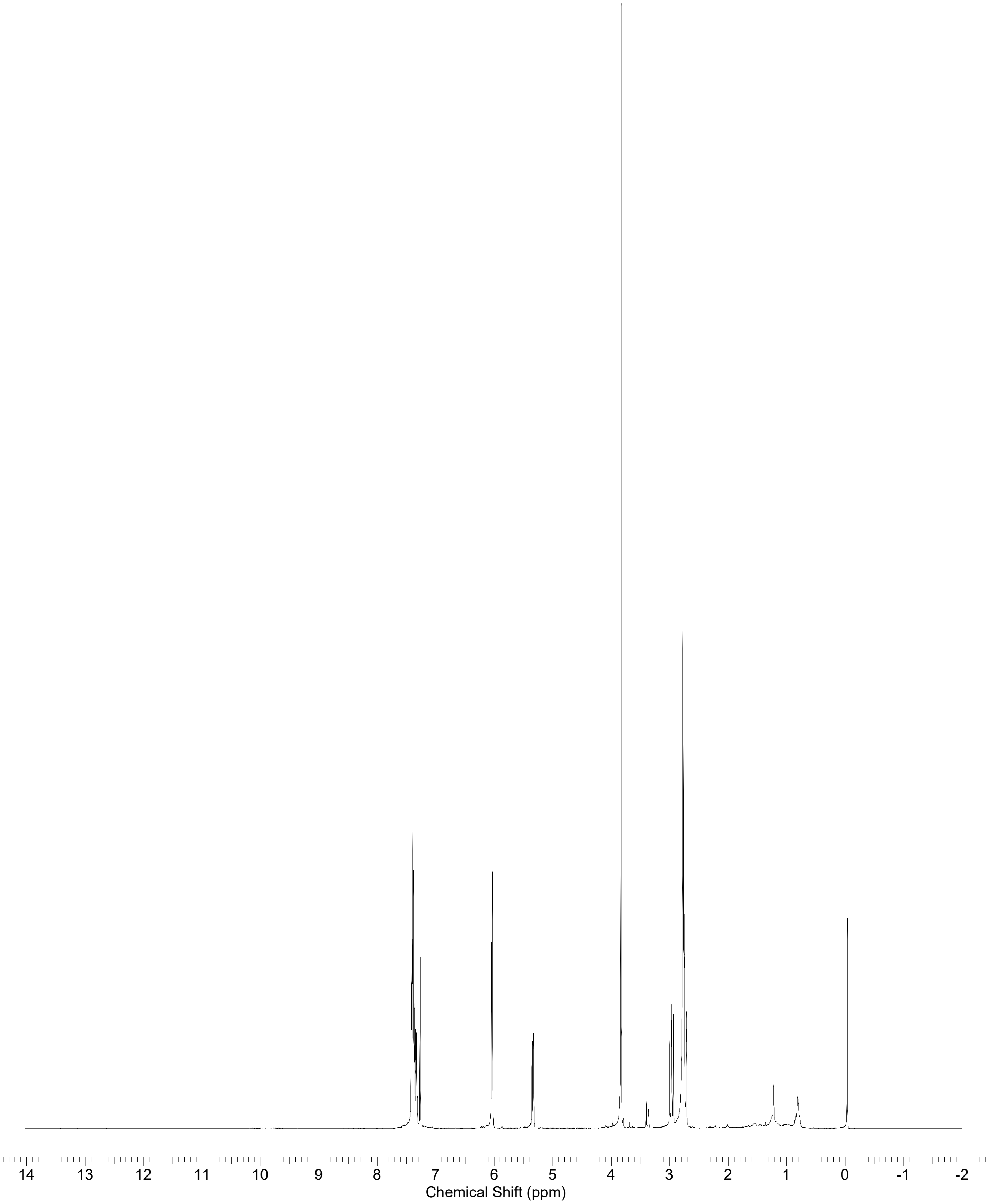

Supplement: Supplementary file 1 [file molecules-21-00417-s001.zip › Supplementary/S4 files/HNMR/1HNMR APN.pdf]
