# Supplementary material for: In Silico Discovery of Potential Uridine-Cytidine Kinase 2 Inhibitors from the Rhizome of Alpinia mutica
Source: Molecules. 2016 Apr 8;21(4):417. doi: 10.3390/molecules21040417 (PMC6274218; doi:10.3390/molecules21040417)

MAKMAL GCMS, JABATAN KIMIA,
FAKULTI SAINS, UPM, SERDANG, SELANGOR
DIMS 2010 SHIMADZU

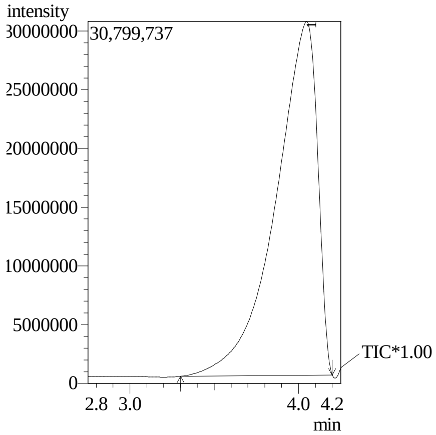

Sample Name : IB 17
Sample ID : GS41647ibrahimMalami

DIMS Sample Information

Data Acquired by	: Admin
Acquisiton Date	: 1/12/2016 11:14:46 AM
Data File	: C:\GCMSsolution\Data\KR_kartineeKassim\4029d-kr-IB17-1.qgd
Method File	: C:\GCMSsolution\Data\KR_KartineeKassim\DI2010-T.qgm
Report File	: DIpotrait1.qgr
Tuning File	: C:\GCMSsolution\System\Tune1\090116EIf1zb5ms.qgt
Modified by	: Admin
Modified	: 1/12/2016 11:46:52 AM

DIMS Library

<< Target >>
Line#:1 R.Time:4.050(Scan#:463) MassPeaks:101 BasePeak:166.05(6612121)
RawMode:Averaged 4.042-4.058(462-464) BG Mode:Calc. from Peak

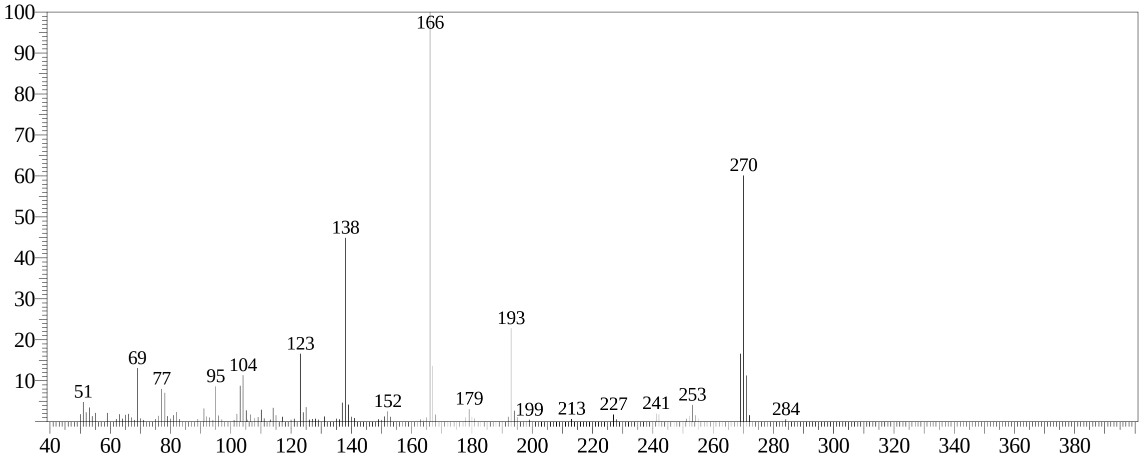

Hit#:1 Entry:90499 Library:NIST08.LIB
SI:81 Formula:C16H14O4 CAS:28143-82-0 MolWeight:270 RetIndex:2440
CompName:2-Propen-1-one, 1-(2,4-dihydroxy-5-methoxyphenyl)-3-phenyl-, (E)- $$ Chalcone, 2',4'-dihydroxy-5'-methoxy- $$ Flemi c

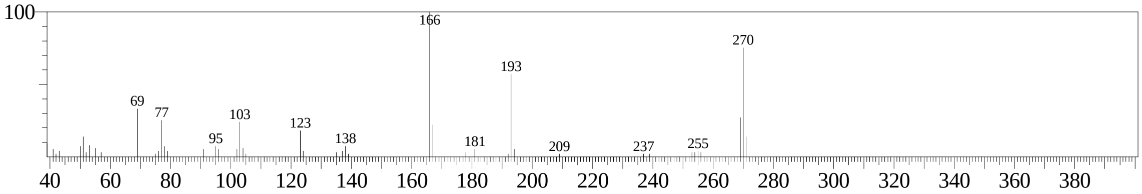

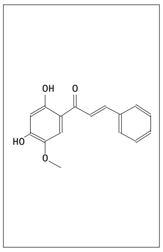

Hit#:2 Entry:90506 Library:NIST08.LIB
SI:77 Formula:C16H14O4 CAS:18956-15-5 MolWeight:270 RetIndex:2440
CompName:2-Propen-1-one, 1-(2,6-dihydroxy-4-methoxyphenyl)-3-phenyl-, (E)- $$ Chalcone, 2',6'-dihydroxy-4'-methoxy- $$ Pinostro

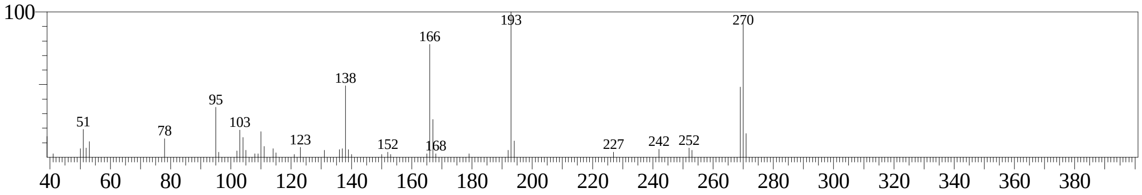

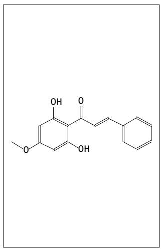

Hit#:3 Entry:90661 Library:NIST08.LIB
SI:66 Formula:C17H18O3 CAS:55670-26-3 MolWeight:270 RetIndex:2313
CompName:2H-1-Benzopyran-7-ol, 3,4-dihydro-5-methoxy-6-methyl-2-phenyl- $$ 5-Methoxy-6-methyl-2-phenyl-7-chromanol # $$

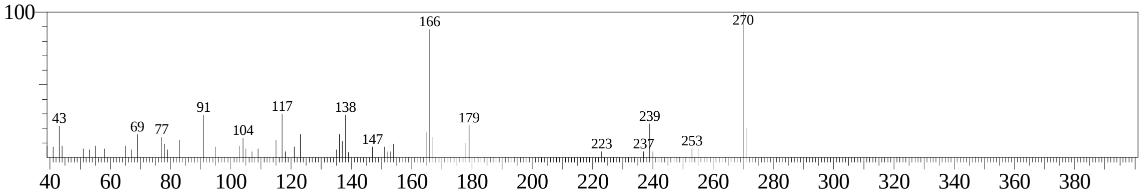

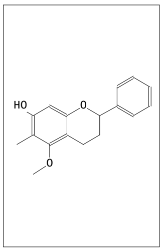

GCMS Mass Table
Line#:1 R.Time:4.0(Scan#:463)
MassPeaks:101 BasePeak:166(6612121)
RawMode:Averaged 4.0-4.1(462-464)
BG Mode:Calc. from Peak

#	m/z	Abs. Int.	Rel. Int.	#	m/z	Abs. Int.	Rel. Int.	#	m/z	Abs. Int.	Rel. Int.
1	50.00	118826	1.80	35	101.05	27635	0.42	69	151.00	82040	1.24
2	51.00	317673	4.80	36	102.05	122474	1.85	70	152.00	163423	2.47
3	52.00	147263	2.23	37	103.05	577505	8.73	71	153.05	78020	1.18
4	53.00	225280	3.41	38	104.05	749951	11.34	72	163.00	37332	0.56
5	54.00	86818	1.31	39	105.05	180664	2.73	73	164.05	28530	0.43
6	55.00	140749	2.13	40	105.60	30103	0.46	74	165.05	64636	0.98
7	59.00	137947	2.09	41	106.60	114605	1.73	75	166.05	6612121	100.00
8	62.00	39048	0.59	42	108.00	55384	0.84	76	167.00	897728	13.58
9	63.00	119470	1.81	43	109.00	72702	1.10	77	168.00	115112	1.74
10	64.00	48833	0.74	44	110.05	190648	2.88	78	178.00	67616	1.02
11	65.00	106369	1.61	45	111.05	52011	0.79	79	179.00	202129	3.06
12	66.00	125330	1.90	46	113.05	32238	0.49	80	180.00	78257	1.18
13	67.00	68438	1.04	47	114.05	223832	3.39	81	181.00	52914	0.80
14	68.05	27298	0.41	48	115.00	104377	1.58	82	192.00	77831	1.18
15	69.00	863679	13.06	49	117.05	79440	1.20	83	193.00	1510411	22.84
16	70.00	49436	0.75	50	120.00	30018	0.45	84	194.00	178177	2.69
17	71.00	27471	0.42	51	121.00	46810	0.71	85	195.00	71766	1.09
18	75.00	39257	0.59	52	123.00	1095880	16.57	86	199.00	28622	0.43
19	76.05	91406	1.38	53	124.00	150282	2.27	87	213.00	40332	0.61
20	77.00	528326	7.99	54	125.00	233254	3.53	88	227.00	114552	1.73
21	78.05	463403	7.01	55	126.05	31354	0.47	89	228.05	34049	0.51
22	79.00	85833	1.30	56	127.05	38801	0.59	90	241.05	131510	1.99
23	80.00	44636	0.68	57	128.05	48114	0.73	91	242.05	117594	1.78
24	81.00	102542	1.55	58	129.05	29042	0.44	92	251.05	48384	0.73
25	82.05	153286	2.32	59	131.05	82264	1.24	93	252.05	92607	1.40
26	82.95	39777	0.60	60	135.00	48803	0.74	94	253.05	266495	4.03
27	89.00	43005	0.65	61	136.00	34269	0.52	95	254.05	104510	1.58
28	91.05	210985	3.19	62	137.00	305505	4.62	96	255.05	53937	0.82
29	92.05	82742	1.25	63	138.05	2963225	44.82	97	269.10	1093182	16.53
30	93.00	66019	1.00	64	139.00	275922	4.17	98	270.10	3972119	60.07
31	94.05	27668	0.42	65	140.05	78822	1.19	99	271.10	745937	11.28
32	95.00	568392	8.60	66	141.05	55932	0.85	00	272.10	101672	1.54
33	96.00	95634	1.45	67	149.00	36782	0.56	01	284.15	34538	0.52
34	97.00	35533	0.54	68	150.00	26607	0.40				

t IB 17 C:\GCMSsolution\Data\KR_kartineeKassim\4029c
Group #1

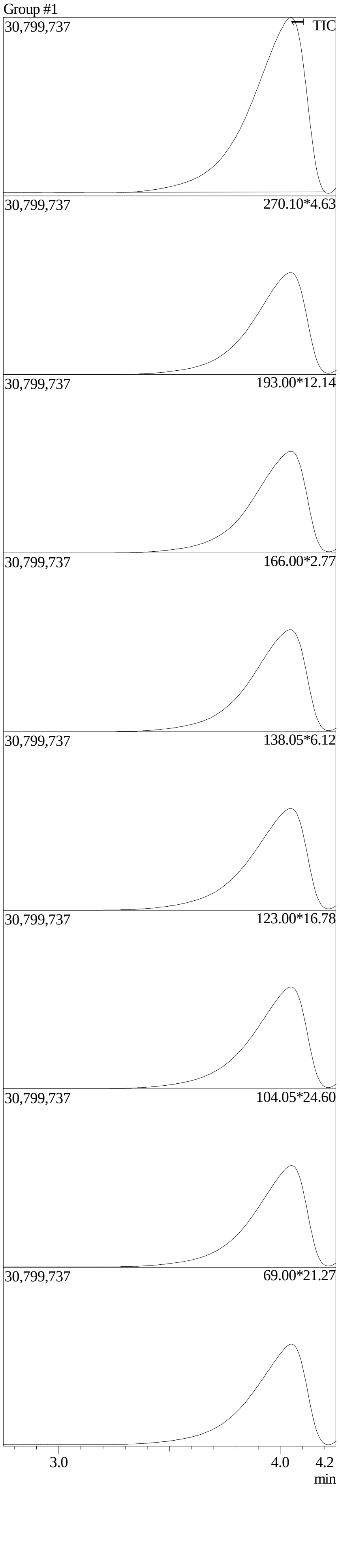

Supplement: Supplementary file 1 [file molecules-21-00417-s001.zip › Supplementary/S5 files/DI-MS for Alpinetin.pdf]
