# Supplementary material for: In Silico Discovery of Potential Uridine-Cytidine Kinase 2 Inhibitors from the Rhizome of Alpinia mutica
Source: Molecules. 2016 Apr 8;21(4):417. doi: 10.3390/molecules21040417 (PMC6274218; doi:10.3390/molecules21040417)

DIMS QP5050A SHIMADZU
MAKMAL GCMS G37
BANGUNAN SAINS FIZIK
JABATAN KIMIA
FAKULTI SAINS
UPM SERDANG

Sample Name : IB 08
Sample ID : GS41647ibrahimMalami

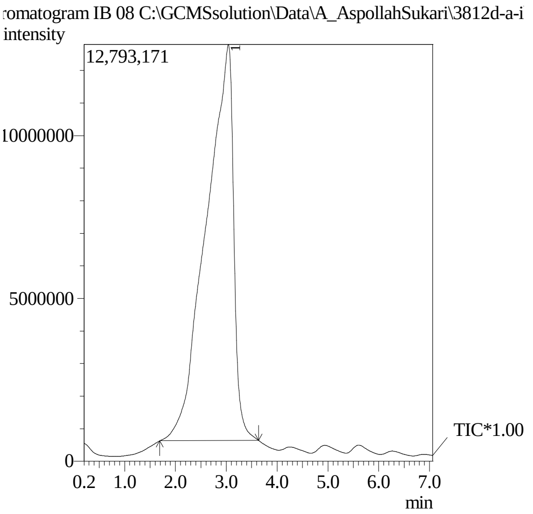

DIMS Sample Information

Data Acquired by	: Admin
Acquisiton Date	: 4/12/2015 12:03:06 PM
Sample Type	: Unknown
Level #	: 1
Sample Name	: IB 08
Sample ID	: GS41647ibrahimMalami
IS Amount	: [1]=1
Sample Amount	: 1
Dilution Factor	: 1
Vial #	: 1
Injection Volume	: 4
Data File	: C:\GCMSsolution\Data\A_AspollahSukari\3812d-a-ib08-1.qgd
Method File	: D:\Data2015\A_AspollahHjSukari\DI50-A.qgm
Report File	: D:\Data2015\A_AspollahHjSukari\dipotrait.qgr
Tuning File	: C:\GCMSsolution\System\Tune1\040415EIf1zbffap.qgt
Modified by	: Admin
Modified	: 4/11/2015 9:44:14 PM

DIMS Library

<< Target >>
Line#:1 R.Time:3.042(Scan#:342) MassPeaks:146 BasePeak:207.05(1960392)
RawMode:Averaged 3.033-3.050(341-343) BG Mode:Calc. from Peak

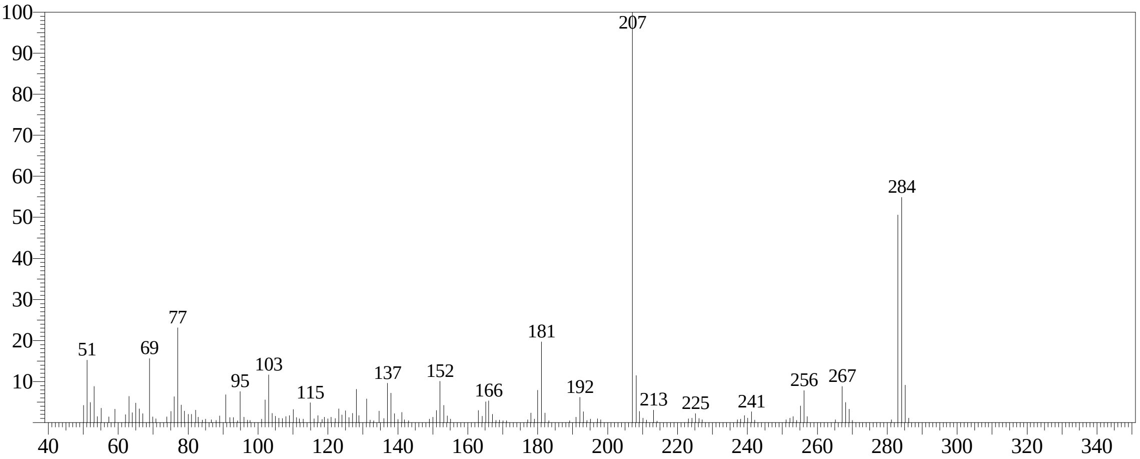

Hit#:1 Entry:100921 Library:NIST08.LIB
SI:63 Formula:C20H16N2 CAS:0-00-0 MolWeight:284 RetIndex:2648
CompName:Benzaldehyde,(diphenylmethylidene)hydrazone

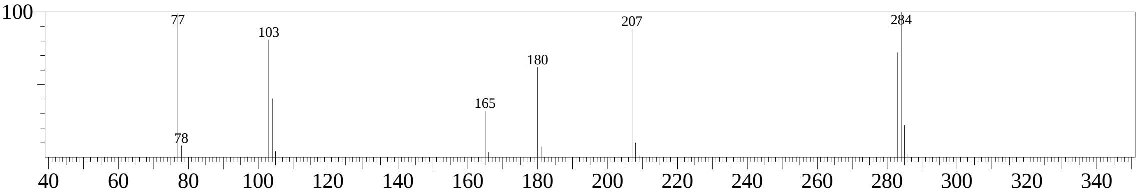

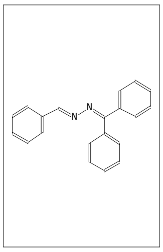

Hit#:2 Entry:113726 Library:NIST08.LIB
SI:60 Formula:C21H18O2 CAS:4376-83-4 MolWeight:302 RetIndex:2631
CompName:1-Propanone, 3-(2-hydroxyphenyl)-1,3-diphenyl- $$ Propiophenone, 3-(o-hydroxyphenyl)-3-phenyl- $$ 3-(2-Hydroxyphenyl

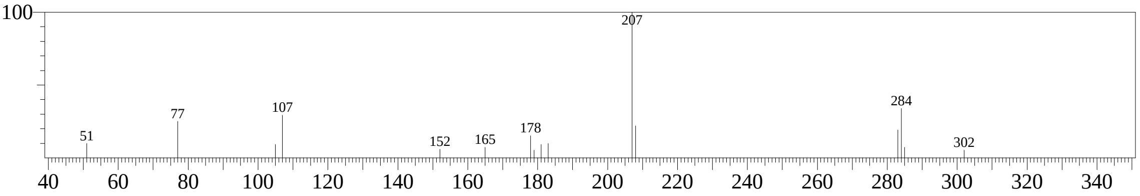

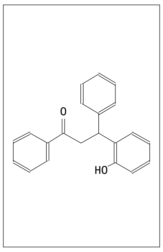

Hit#:3 Entry:100949 Library:NIST08.LIB
SI:59 Formula:C21H16O CAS:849-01-4 MolWeight:284 RetIndex:2460
CompName:Acrylophenone, 3,3-diphenyl- $$ Chalcone, .beta.-phenyl- $$ 2-Propen-1-one, 1,3,3-triphenyl- $$ 1,3,3-Triphenyl-2-propen-

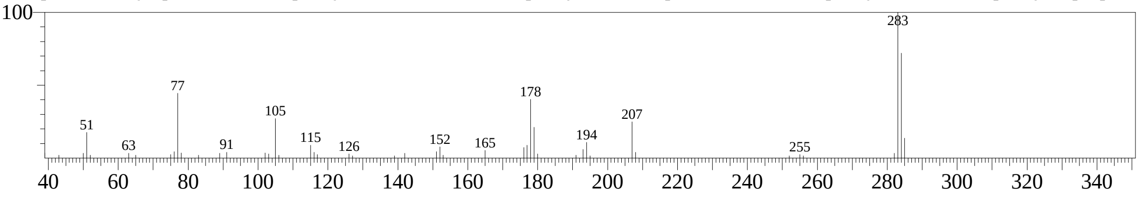

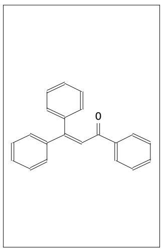

GCMS Mass Table
Line#:1   R.Time:3.0(Scan#:342)
MassPeaks:146   BasePeak:207(1960392)
RawMode:Averaged 3.0-3.1(341-343)
BG Mode:Calc. from Peak

#	m/z	Abs. Int.	Rel. Int.	#	m/z	Abs. Int.	Rel. Int.	#	m/z	Abs. Int.	Rel. Int.
1	50.05	82506	4.21	50	110.05	62893	3.21	99	179.10	17287	0.88
2	51.10	298380	15.22	51	111.00	24802	1.27	100	179.95	154067	7.86
3	52.05	96392	4.92	52	111.85	19107	0.97	101	181.10	385705	19.67
4	53.10	173461	8.85	53	112.90	16541	0.84	102	182.10	45449	2.32
5	54.05	29086	1.48	54	114.95	94367	4.81	103	183.20	9932	0.51
6	55.10	68412	3.49	55	116.05	17704	0.90	104	189.10	8701	0.44
7	57.30	27549	1.41	56	117.10	33036	1.69	105	191.00	25670	1.31
8	59.05	63698	3.25	57	118.35	14856	0.76	106	192.10	121039	6.17
9	62.05	37587	1.92	58	119.00	25430	1.30	107	193.10	52505	2.68
10	63.05	126169	6.44	59	119.95	18570	0.95	108	194.10	11469	0.59
11	64.05	47525	2.42	60	120.90	26295	1.34	109	195.05	17019	0.87
12	65.05	92718	4.73	61	122.05	19134	0.98	110	197.10	19007	0.97
13	66.00	65593	3.35	62	123.05	65100	3.32	111	198.05	13732	0.70
14	67.05	42251	2.16	63	124.05	36166	1.84	112	207.05	1960392	100.00
15	69.00	305638	15.59	64	125.05	56834	2.90	113	208.15	225334	11.49
16	69.90	28122	1.43	65	126.00	24978	1.27	114	209.15	53063	2.71
17	70.75	17860	0.91	66	127.00	43908	2.24	115	210.15	21393	1.09
18	73.90	27263	1.39	67	128.10	158457	8.08	116	211.15	12997	0.66
19	75.05	53113	2.71	68	128.85	33295	1.70	117	213.10	59740	3.05
20	76.05	124382	6.34	69	131.05	113479	5.79	118	214.15	8181	0.42
21	77.00	452349	23.07	70	132.10	12848	0.66	119	223.15	17961	0.92
22	78.00	83968	4.28	71	133.05	9402	0.48	120	224.15	21881	1.12
23	78.95	54852	2.80	72	134.65	54546	2.78	121	225.10	42474	2.17
24	80.00	39401	2.01	73	136.00	19283	0.98	122	226.10	19780	1.01
25	81.00	40317	2.06	74	137.00	188595	9.62	123	227.10	14110	0.72
26	82.10	58982	3.01	75	138.00	140874	7.19	124	237.15	14007	0.71
27	82.85	25572	1.30	76	139.00	42764	2.18	125	238.05	16550	0.84
28	84.05	11711	0.60	77	140.05	14762	0.75	126	239.15	32915	1.68
29	85.00	16236	0.83	78	141.10	48745	2.49	127	240.10	21116	1.08
30	86.60	13949	0.71	79	141.90	14170	0.72	128	241.15	52145	2.66
31	88.00	10510	0.54	80	143.10	9163	0.47	129	242.10	11664	0.59
32	89.00	31644	1.61	81	149.05	16378	0.84	130	251.05	14499	0.74
33	90.80	133053	6.79	82	150.00	25789	1.32	131	252.15	21917	1.12
34	91.95	24067	1.23	83	151.00	58696	2.99	132	253.10	28901	1.47
35	92.95	24049	1.23	84	152.05	196849	10.04	133	254.10	11376	0.58
36	94.10	8426	0.43	85	153.10	82420	4.20	134	255.15	79976	4.08
37	94.85	148407	7.57	86	154.10	32026	1.63	135	256.15	153216	7.82
38	96.00	26655	1.36	87	155.05	17293	0.88	136	257.10	29628	1.51
39	96.95	11664	0.59	88	163.00	58084	2.96	137	265.15	13068	0.67
40	97.70	10700	0.55	89	164.10	30200	1.54	138	267.10	172768	8.81
41	101.00	16529	0.84	90	165.10	98781	5.04	139	268.10	96324	4.91
42	102.05	108602	5.54	91	165.95	104157	5.31	140	269.10	65039	3.32
43	103.05	228086	11.63	92	167.05	39724	2.03	141	270.00	10174	0.52
44	104.05	44852	2.29	93	168.05	10362	0.53	142	281.20	13386	0.68
45	105.00	29924	1.53	94	169.10	12619	0.64	143	283.05	991696	50.59
46	106.00	21114	1.08	95	170.10	9661	0.49	144	284.10	1076371	54.91
47	106.95	19575	1.00	96	171.05	9439	0.48	145	285.15	179097	9.14
48	108.00	29800	1.52	97	177.15	14043	0.72	146	286.15	22113	1.13
49	109.00	34422	1.76	98	178.05	46147	2.35				

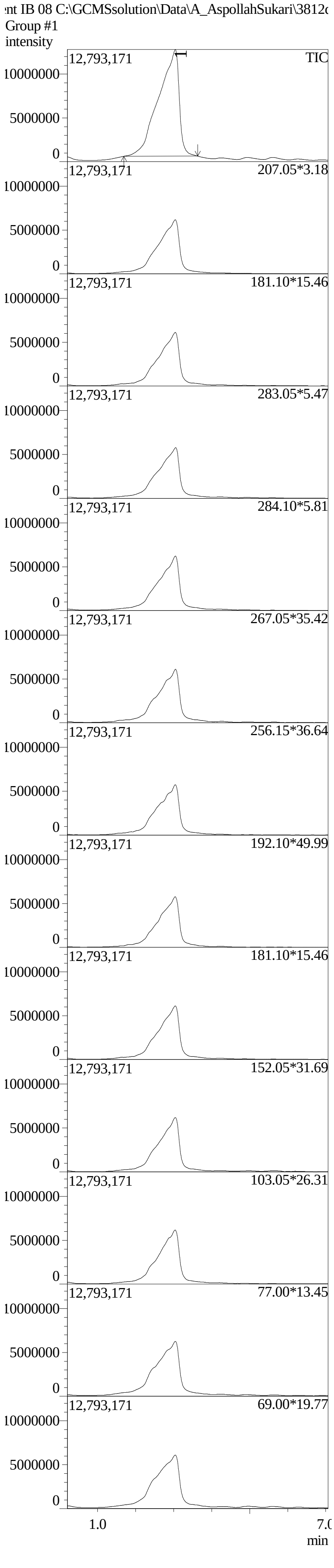

Supplement: Supplementary file 1 [file molecules-21-00417-s001.zip › Supplementary/S5 files/DI-MS for Flavokawain B.pdf]
