# Supplementary figures and images for: In Silico Discovery of Potential Uridine-Cytidine Kinase 2 Inhibitors from the Rhizome of Alpinia mutica
Source: Molecules. 2016 Apr 8;21(4):417. doi: 10.3390/molecules21040417 (PMC6274218; doi:10.3390/molecules21040417)

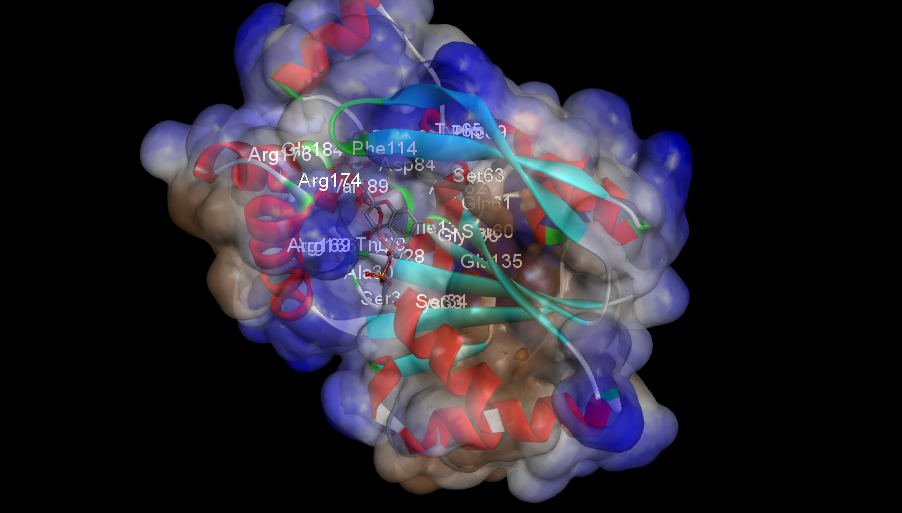

Supplement: Supplementary file 1 [file molecules-21-00417-s001.zip › Supplementary/S2 files/1UDW+APN/1UDW hydrophobicity.png]

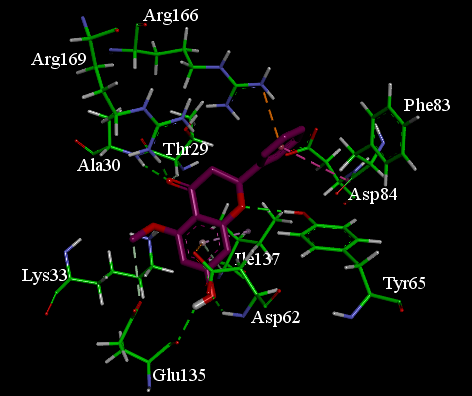

Supplement: Supplementary file 1 [file molecules-21-00417-s001.zip › Supplementary/S2 files/1UDW+APN/1UDW interraction.png]

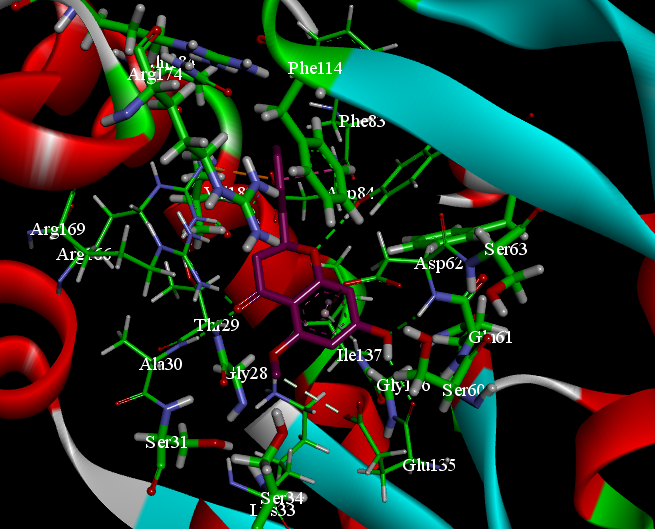

Supplement: Supplementary file 1 [file molecules-21-00417-s001.zip › Supplementary/S2 files/1UDW+APN/1UDW interraction2.png]

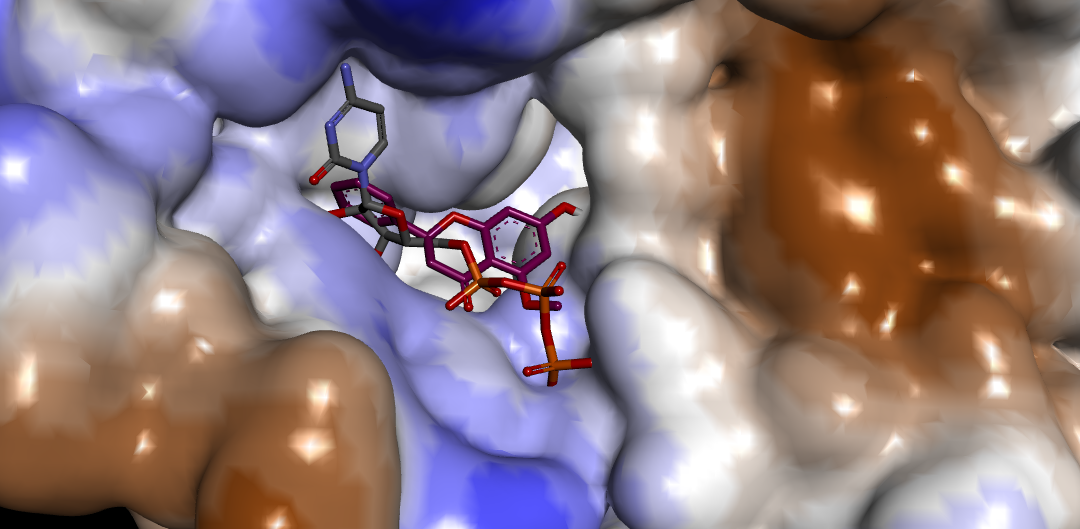

Supplement: Supplementary file 1 [file molecules-21-00417-s001.zip › Supplementary/S2 files/1UDW+APN/1UDW surface binding pocket.png]

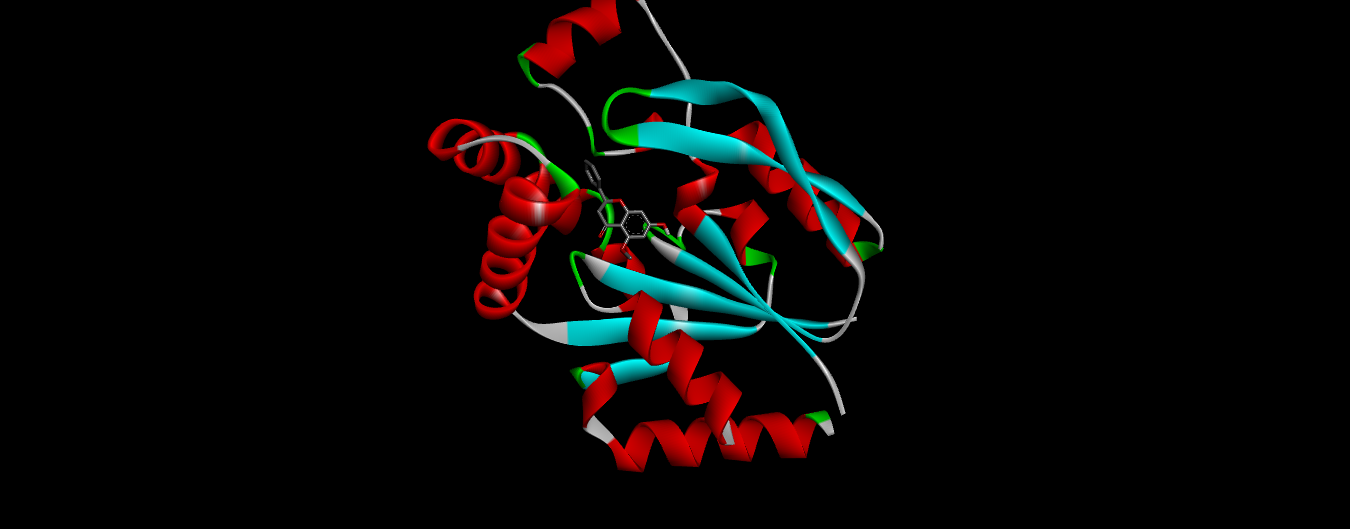

Supplement: Supplementary file 1 [file molecules-21-00417-s001.zip › Supplementary/S2 files/1UDW+APN/1UDW+Lig.png]

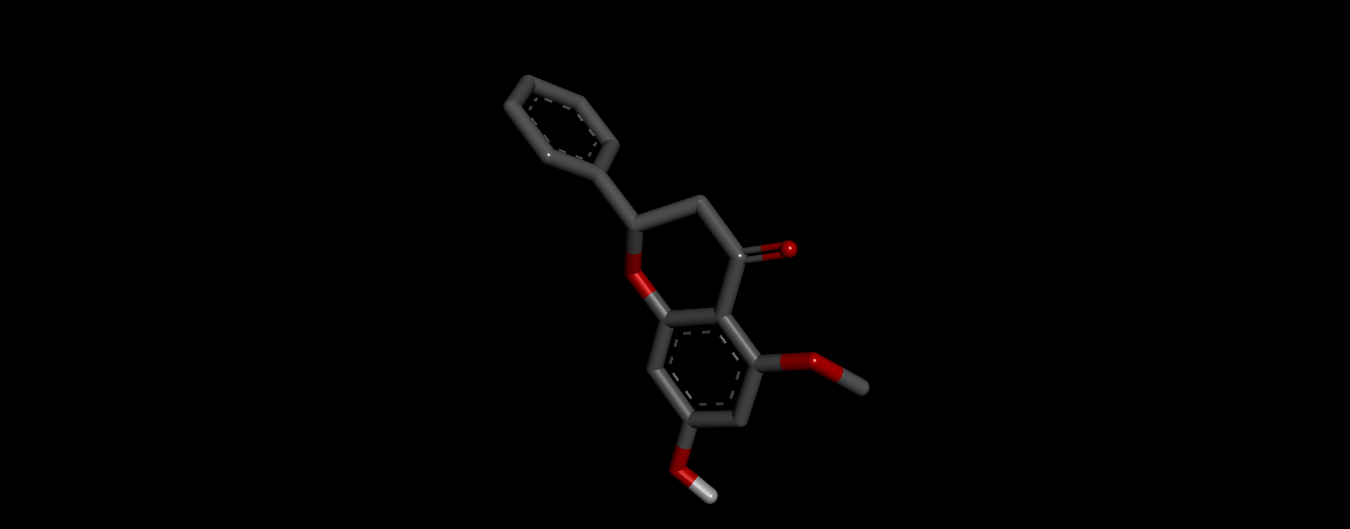

Supplement: Supplementary file 1 [file molecules-21-00417-s001.zip › Supplementary/S2 files/1UDW+APN/alpinetin_lig_1.png]

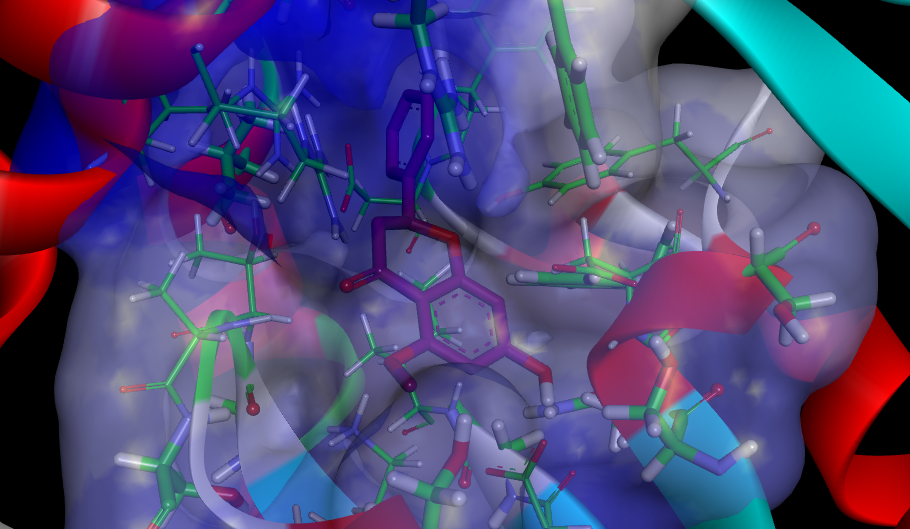

Supplement: Supplementary file 1 [file molecules-21-00417-s001.zip › Supplementary/S2 files/1UDW+APN/hydrophobicity.png]

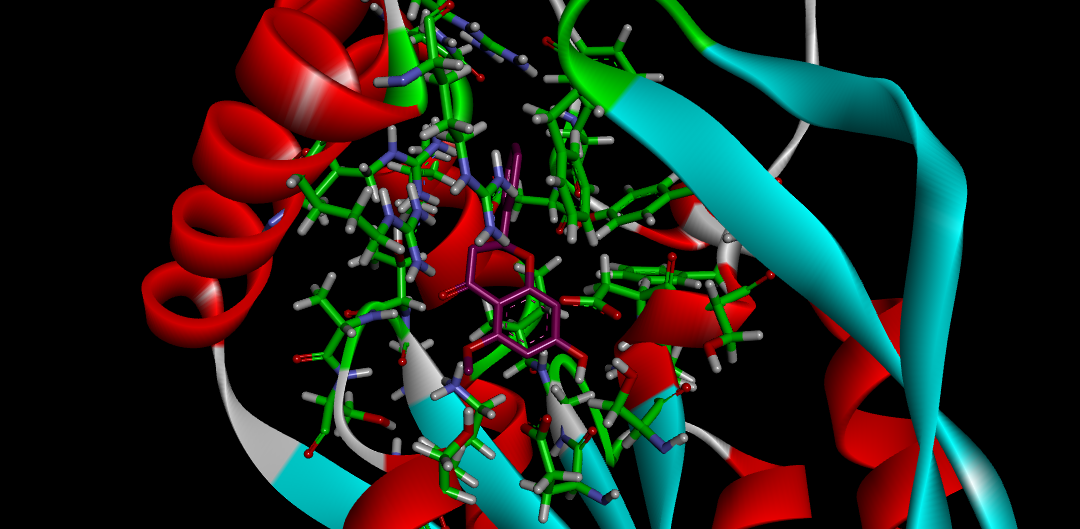

Supplement: Supplementary file 1 [file molecules-21-00417-s001.zip › Supplementary/S2 files/1UDW+APN/Lig active site.png]

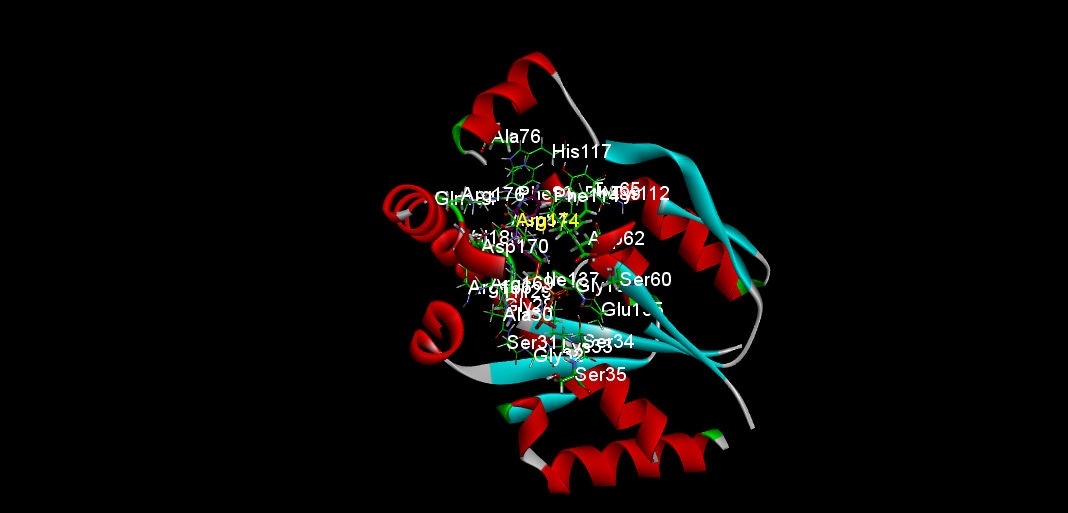

Supplement: Supplementary file 1 [file molecules-21-00417-s001.zip › Supplementary/S2 files/1UDW+CTP/1UDW interraction.png]

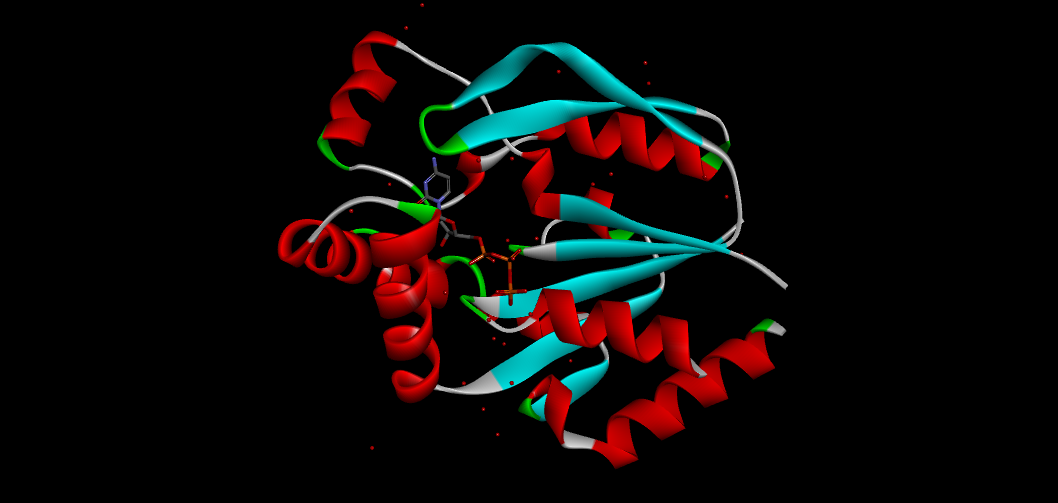

Supplement: Supplementary file 1 [file molecules-21-00417-s001.zip › Supplementary/S2 files/1UDW+CTP/1UDW.png]

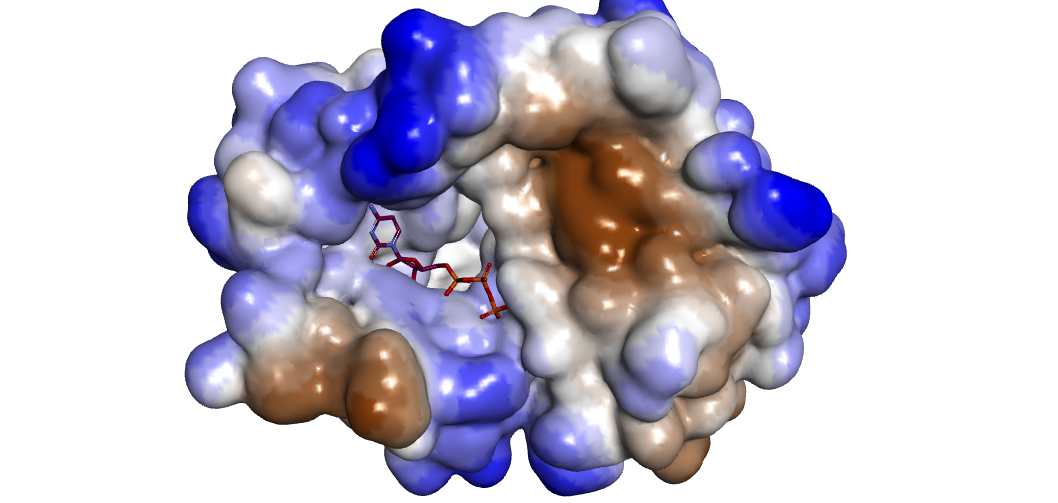

Supplement: Supplementary file 1 [file molecules-21-00417-s001.zip › Supplementary/S2 files/1UDW+CTP/1UDW+CTP surface binding pocket.png]

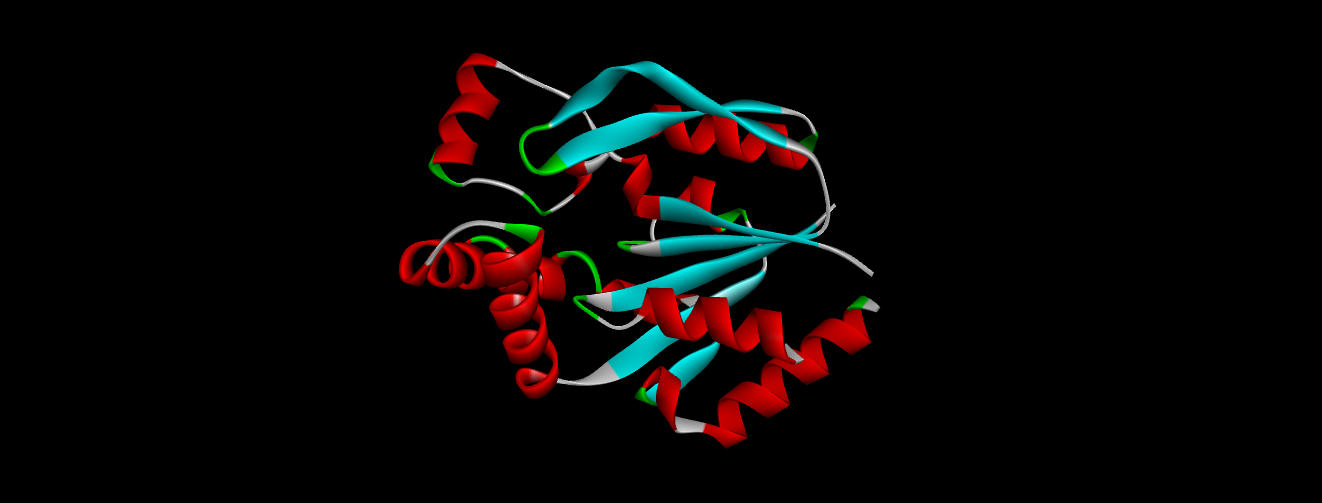

Supplement: Supplementary file 1 [file molecules-21-00417-s001.zip › Supplementary/S2 files/1UDW+CTP/1UDW2.png]

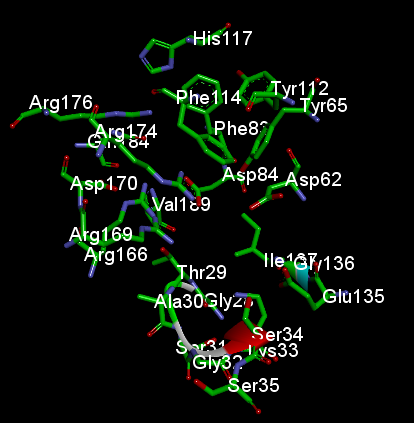

Supplement: Supplementary file 1 [file molecules-21-00417-s001.zip › Supplementary/S2 files/1UDW+CTP/active site.png]

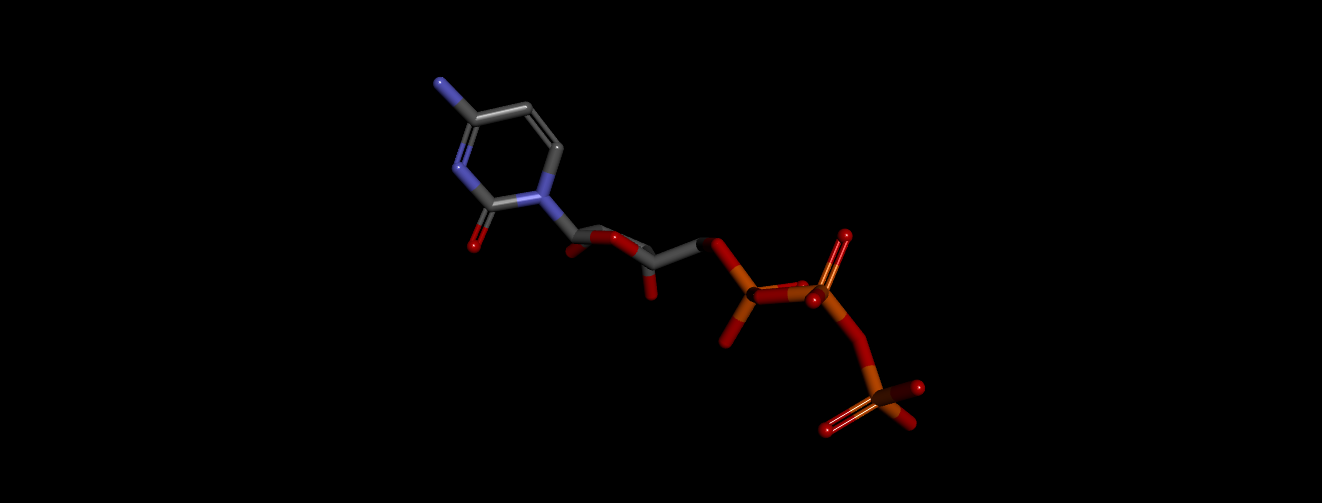

Supplement: Supplementary file 1 [file molecules-21-00417-s001.zip › Supplementary/S2 files/1UDW+CTP/CTP.png]

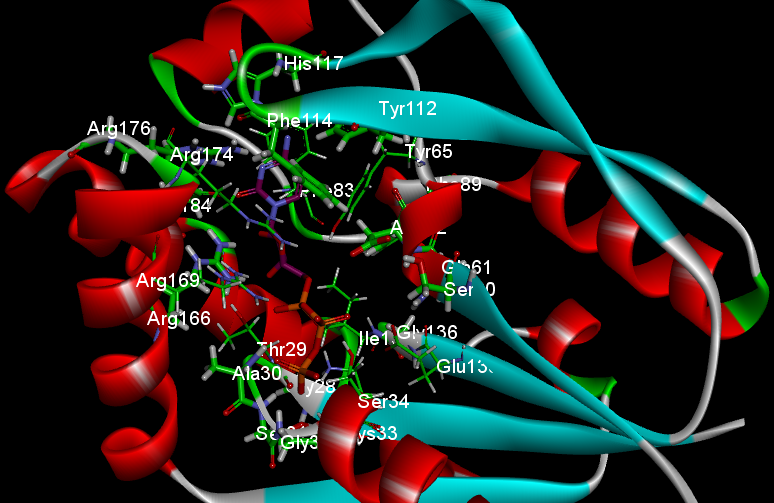

Supplement: Supplementary file 1 [file molecules-21-00417-s001.zip › Supplementary/S2 files/1UDW+CTP/CTP+1UDW interraction.png]

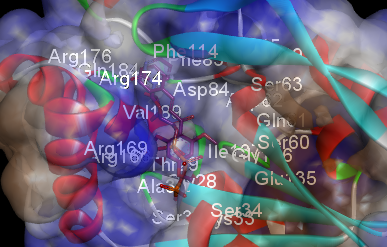

Supplement: Supplementary file 1 [file molecules-21-00417-s001.zip › Supplementary/S2 files/1UDW+FKB/1UDW hydrophobicity.png]

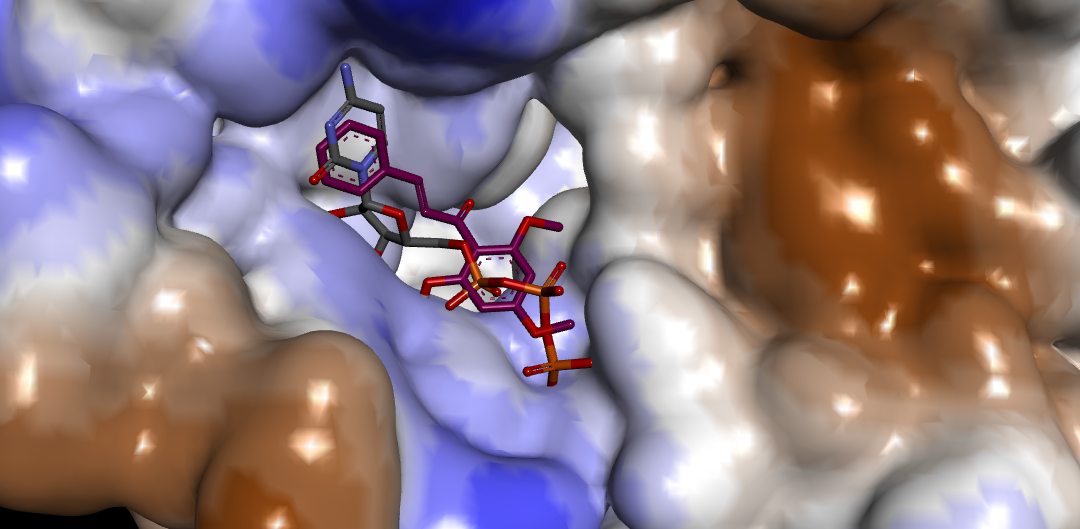

Supplement: Supplementary file 1 [file molecules-21-00417-s001.zip › Supplementary/S2 files/1UDW+FKB/1UDW surface binding pocket.png]

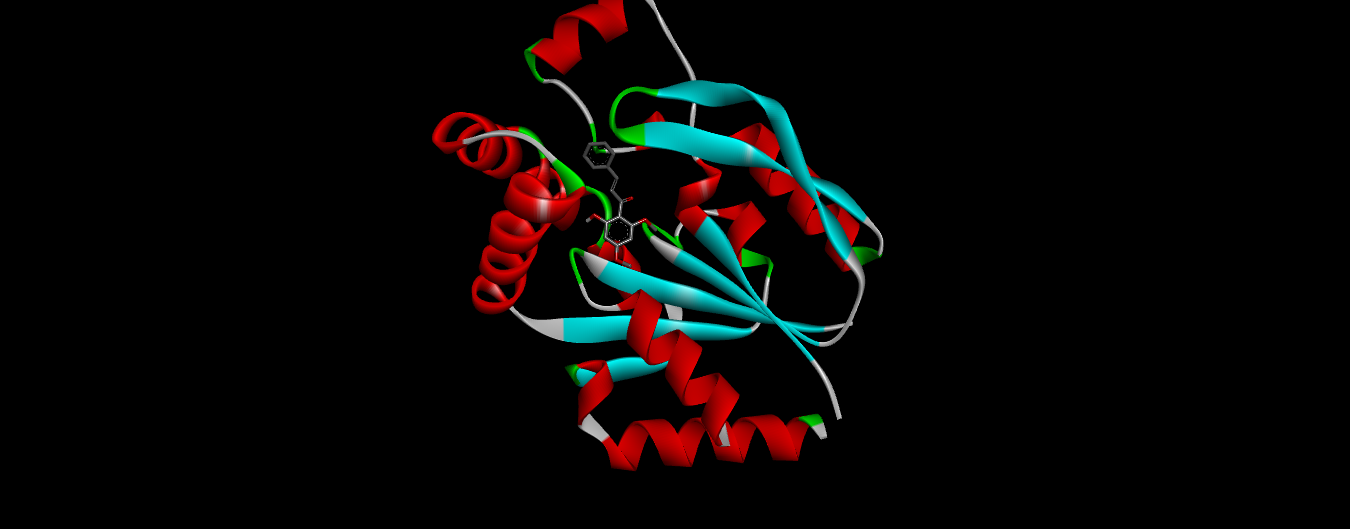

Supplement: Supplementary file 1 [file molecules-21-00417-s001.zip › Supplementary/S2 files/1UDW+FKB/1UDW+lig.png]

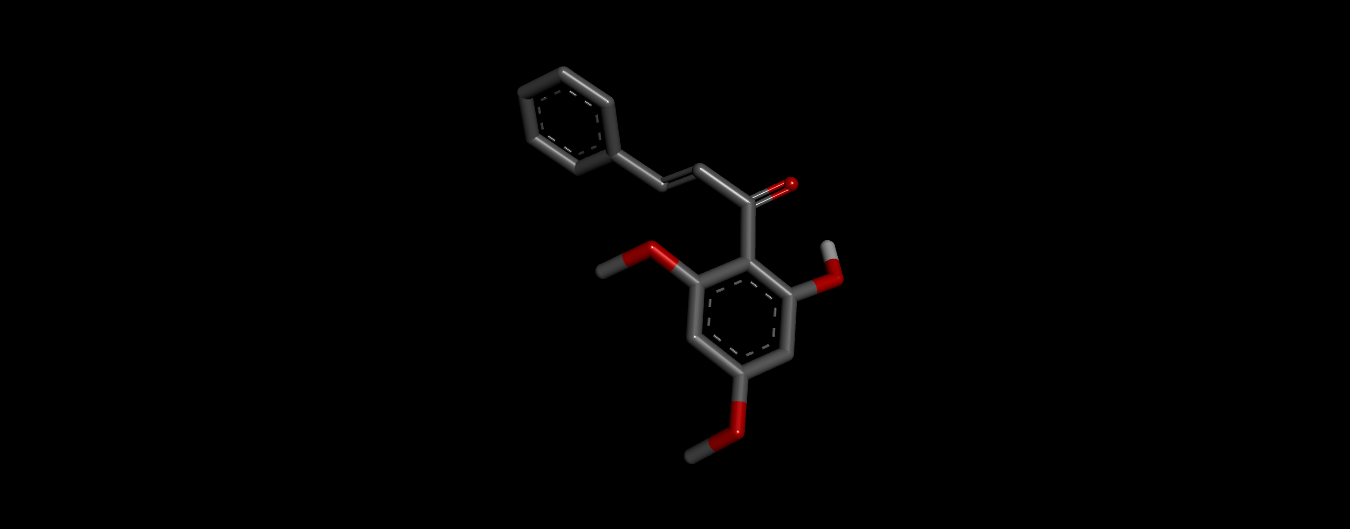

Supplement: Supplementary file 1 [file molecules-21-00417-s001.zip › Supplementary/S2 files/1UDW+FKB/FKB_lig_2.png]

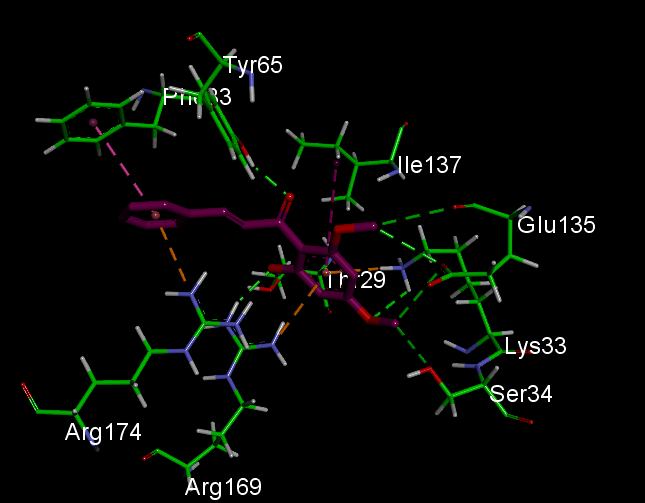

Supplement: Supplementary file 1 [file molecules-21-00417-s001.zip › Supplementary/S2 files/1UDW+FKB/FKB+1UDW interraction.png]

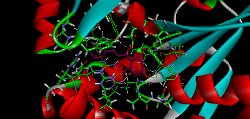

Supplement: Supplementary file 1 [file molecules-21-00417-s001.zip › Supplementary/S2 files/1UDW+FKB/FKB+1UDW interraction2.png]

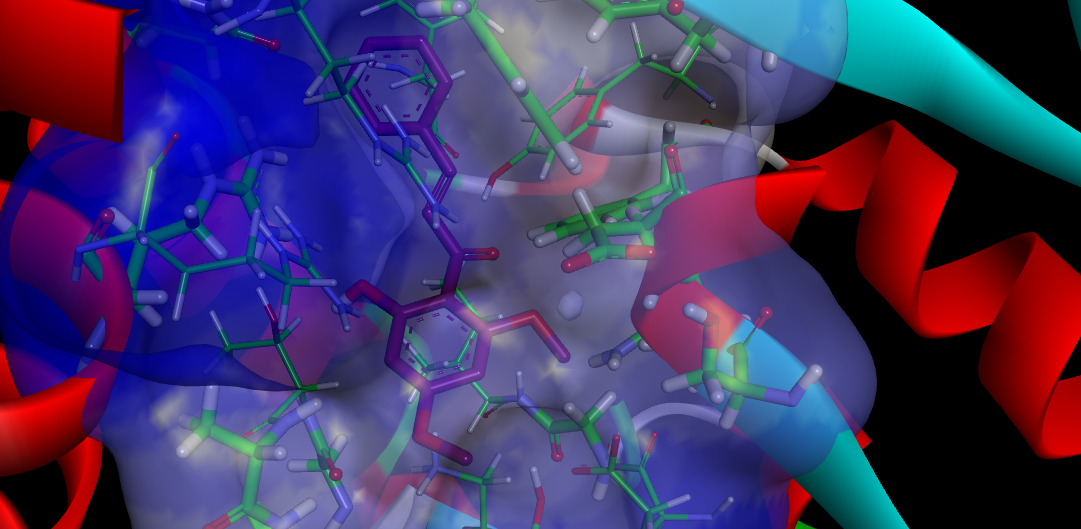

Supplement: Supplementary file 1 [file molecules-21-00417-s001.zip › Supplementary/S2 files/1UDW+FKB/hydrophobicity.png]
